# Lycorine Attenuates Autophagy in Osteoclasts via an Axis of mROS/TRPML1/TFEB to Reduce LPS-Induced Bone Loss

**DOI:** 10.1155/2019/8982147

**Published:** 2019-10-08

**Authors:** Hyun-Jung Park, Malihatosadat Gholam-Zadeh, Jae-Hee Suh, Hye-Seon Choi

**Affiliations:** ^1^Department of Biological Sciences, University of Ulsan, Ulsan 44610, Republic of Korea; ^2^Department of Pathology, Ulsan University Hospital, Ulsan 44030, Republic of Korea

## Abstract

Lycorine, a plant alkaloid, exhibits anti-inflammatory activity by acting in macrophages that share precursor cells with osteoclasts (OCs). We hypothesized that lycorine might decrease bone loss by acting in OCs after lipopolysaccharide (LPS) stimulation, since OCs play a main role in LPS-induced bone loss. Microcomputerized tomography (*μ*CT) analysis revealed that lycorine attenuated LPS-induced bone loss in mice. *In vivo* tartrate-resistant acid phosphatase (TRAP) staining showed that increased surface area and number of OCs in LPS-treated mice were also decreased by lycorine treatment, suggesting that OCs are responsible for the bone-sparing effect of lycorine. *In vitro*, the increased number and activity of OCs induced by LPS were reduced by lycorine. Lycorine also decreased LPS-induced autophagy in OCs by evaluation of decreased lipidated form of microtubule-associated proteins 1A/1B light chain 3B (LC3) (LC3II) and increased sequestosome 1 (p62). Lycorine attenuated oxidized transient receptor potential cation channel, mucolipin subfamily (TRPML1) by reducing mitochondrial reactive oxygen species (mROS) and decreased transcription factor EB (TFEB) nuclear translocation. Lycorine reduced the number and activity of OCs by decreasing autophagy in OCs via an axis of mROS/TRPML1/TFEB. Collectively, lycorine protected against LPS-induced bone loss by acting in OCs. Our data highlight the therapeutic potential of lycorine for protection against inflammatory bone loss.

## 1. Introduction

Inflammatory osteolysis is caused by the pathogenesis of infectious and inflammatory disease, resulting in irreversible bone erosion. This bone loss has been reported to be due to inflammation that stimulates osteoclasts (OCs) either directly or indirectly by activating osteoblasts/stromal cells [1]. Injection of lipopolysaccharide (LPS) leads to increased eroded surface area by increasing the number of OCs in rat femurs [2]. LPS has been shown to increase the number and activity of OCs, leading to bone loss [3, 4]. Increased OC number by LPS has been reported to be due to the induction of differentiation via reactive oxygen species (ROS) [5] and enhancing OC survival [6, 7]. LPS-induced autophagy has been demonstrated to be responsible for increased OC formation and OC activity [4, 5, 8].

Lycorine, a pyrrolophenanthridine ring-type alkaloid, is extracted from *Lycoris radiata* (Amaryllidaceae), a traditional oriental medicinal herb with multiple biological functions and low toxicity [9]. Lycorine exhibits anti-inflammatory activity [10] with free radical scavenging activity [11] and antiviral activity [12] as well as a selective inhibitory effect on cancer cells at low concentrations [13, 14]. At the molecular level, lycorine inhibits NF-*κ*B activation [15] and decreases LPS-induced proinflammatory mediators by attenuating p38 and STAT activation [16]. Lycorine inhibits autophagy via degradation of HMGB1 [17]. Recently, lycorine was demonstrated to prevent OVX-induced bone loss via suppression of MAPK [18].

We investigated whether lycorine can attenuate LPS-induced bone loss in mice. The present studies assessed the detailed molecular mechanisms of lycorine for inhibitory activity on LPS-induced autophagy in OCs.

## 2. Materials and Methods

### 2.1. Reagents and Antibodies

Lycorine was purchased from Chengdu Biopurity Phytochemicals Ltd. (Chengdu, Sichuan, China). Recombinant mouse M-CSF and RANKL were received from R&D Systems, Inc. (Minneapolis, MN, USA). The kits of RatLaps EIA, osteocalcin EIA, and alkaline phosphatase were obtained from Immunodiagnostic Systems Inc. (Fountain Hills, AZ, USA), Biomedical Technologies Inc. (Stoughton, MA, USA), and BioAssay Systems (Hayward, CA, USA), respectively. LPS, MTT (3-(4,5-dimethylthiazol-2-yl)-2,5-diphenyltetrazolium bromide), leukocyte acid phosphatase (TRAP) kit, toluidine blue, diphenyliodonium (DPI), and bafilomycin A were from Sigma Chemical (St. Louis, MO, USA). *N*-(Biotinoyl)-*N*′-(iodoacetyl) ethylenediamine (BIAM) was purchased from Invitrogen (Carlsbad, CA, USA). Antibodies against MCP-1 for coating and detection were from R&D Systems, Inc. NE-PER nuclear and cytoplasmic extraction reagents were from Pierce (Waltham, MA, USA). Primary antibodies used for analysis in this study were as follows: LC3B (#2775) and lamin B1 (#13435) from Cell Signaling Technology (Danvers, MA, USA), p62 (H00008878-M01) from Abnova (Taipei, Taiwan), TFEB (A303-673A) from Bethyl Laboratories, Inc. (Montgomery, TX, USA), TRPML1 (ACC-081) from Alomone Labs, Ltd. (Jerusalem, Israel), and *β*-actin (A5441) from Sigma Chemical. HRP-conjugated secondary Abs and 2′,7′-dichlorofluorescein diacetate (H_2_DCFDA) were obtained from Thermo Fisher Scientific (Waltham, MA, USA). Small interfering RNA (siRNA) against TRPML1 (sc-44520), scrambled siRNA (scRNA, sc-37007), and Mito-TEMPO were from Santa Cruz Biotechnology (Santa Cruz, CA, USA). M-MLV reverse transcriptase and SYBR Green Real-Time PCR Master Mixes were from Promega (Madison, WI, USA). QIAzol reagent was purchased from QIAGEN (Hilden, Germany). Lipofectamine 3000 and MitoSOX Red were from Invitrogen.

### 2.2. Animals and Study Design

C57BL/6J female mice (10 weeks old) were kept in the pathogen-free animal facility of IRC. All mice were treated in accordance with the guidelines of the Institutional Animal Care and Use Committee (IACUC) of the Immunomodulation Research Center (IRC), University of Ulsan, following the approval by the IACUC of IRC. The approval ID for this study is #HSC-15-011. Animals were randomly divided into the following 4 groups: vehicle control (*n* = 5), vehicle+lycorine (*n* = 5), LPS (*n* = 5), and LPS+lycorine (*n* = 6). Mice were injected with LPS (5 mg/kg, i.p.) in 200 *μ*l phosphate-buffered saline (PBS) once per week for 3 weeks [19]. Lycorine was solubilized with 1 N HCl in PBS and neutralized with sodium hydroxide to reach pH 7.2; mice were treated with lycorine once every two days intraperitoneally in 200 *μ*l PBS (or with PBS as a vehicle) at a dose of 6 mg/kg for 3 weeks. Mice were sacrificed by CO_2_. To analyze bone mineral density (BMD) and microarchitecture, the right femur was scanned in a high-resolution micro-CT (*μ*CT) SkyScan 1176 System (Bruker Micro-CT, Kontich, Belgium) following the methods of Park et al. [4]. Serum collagen-type I fragment (CTX-1) level was measured with commercial RatLaps EIA assay kits. Osteocalcin and alkaline phosphatase (ALP) of serum were assessed using an osteocalcin EIA kit and a colorimetric kinetic determination kit, respectively. Serum MCP-1 was evaluated by sandwich ELISA using the recommended Abs according to the manufacturer's instructions.

### 2.3. OC Formation

At 4-5 weeks of age, C57BL/6J bone marrow cells were isolated from the tibiae and femurs according to a previously described method [20]. Further steps were performed following the methods of Park et al. [4]. After incubation for the indicated times, the cells were fixed in 10% formalin for 10 min and stained for tartrate-resistant acid phosphatase (TRAP). The number of TRAP-positive multinucleated cells (MNCs) (three or more nuclei), area, and maximal diameter of the formed OCs were measured, and the fusion index was presented as the average number of nuclei per TRAP-positive MNC [21].

### 2.4. Cell Viability

Equal numbers of bone marrow-derived macrophages (BMMs) were seeded into each well of a 96-well plate. Cell viability was determined by MTT assay, using MTT (3-(4,5-dimethylthiazol-2-yl)-2,5-diphenyltetrazolium bromide) (0.5 mg/ml) at 37°C for 3 h. After removing MTT and adding 100 *μ*l dimethylformamide (DMSO), the absorbance was measured at 540 nm using a microplate reader.

### 2.5. RNA Isolation and Quantitative Polymerase Chain Reaction (qPCR)

For determining mRNA levels, total RNA was isolated using QIAzol reagent, following the methods of Park et al. [4]. Relative mRNA levels were quantified using a standard curve and normalized to the housekeeping gene 18S rRNA (RPS). Relative gene expression was calculated using the formula 2^-*∆∆*Ct^, where *∆*Ct is the difference in Ct value (threshold of cycle) of the target gene and the gene for normalization (RPS). Primer sequences were 5′-ctccaacaaggtgcttggga-3′ and 5′-gaagcagtagatagtcgcca-3′ (calcitonin receptor), 5′-gaccaccttggcaatgtctctg-3′ and 5′-tggctgaggaagtcatctgagttg-3′ (TRAP), 5′-gtgggtgttcaagtttctgc-3′ and 5′-ggtgagtcttcttccatagc-3′ (cathepsin K), 5′-agacgtggtttaggaatgcagctc-3′ and 5′-tcctccatgaacaaacagttccaa-3′ (DC-STAMP), 5′-ttcagttgctatccaggactcgga-3′ and 5′-gcatgtcatgtaggtgagaaatgtgctca-3′ (ATP6v0d2), 5′-atctacctgggctattgcttctgtg-3′ and 5′-tgtcgttccgttgatgagtg-3′ (TRPML1), and 5′-atcagagagttgaccgcagttg-3′ and 5′-aatgaaccgaagcacaccatag-3′ (RPS).

### 2.6. Bone Resorption

To evaluate the ability of OCs to form pits, mature OCs were cultured on dentine slices [22]. To generate mature OCs, preosteoclast was cultured with M-CSF and RANKL in 60 mm dishes. After 3-4 days, detached mature OCs and seeded equal numbers of cells on top of dentine slices. The cells were incubated with M-CSF (30 ng/ml) and LPS (50 ng/ml) in the presence or absence of lycorine (1.6 *μ*M) for 4 days. Further steps followed the methods of Park et al. [4].

### 2.7. Determination of Intracellular and Mitochondrial Reactive Oxygen Species (ROS)

Intracellular ROS and mitochondria-generated ROS were detected using the fluorescent probe 2′,7′-dichlorofluorescein diacetate (H_2_DCFDA) and MitoSOX Red mitochondrial superoxide indicator, respectively. BMMs were prepared and incubated with M-CSF and RANKL for 40 h, washed thoroughly, incubated further for the indicated periods with LPS in the presence of M-CSF, harvested, suspended in PBS, loaded with H_2_DCFDA or MitoSOX Red, and incubated at 37°C for 30 min. Intracellular and mitochondria-generated ROS were measured by FACSCalibur flow cytometer (Becton Dickinson, Franklin Lakes, NJ, USA).

### 2.8. Detection of Oxidized TRPML1 by Carboxymethylation

OCs were generated from pre-OCs by treatment with M-CSF (30 ng/ml) and LPS (50 ng/ml) in the presence or absence of lycorine (1.6 *μ*M) for 2 d. The medium was removed, and the cells were frozen rapidly in liquid nitrogen. The frozen cells were transferred to 100 *μ*M *N*-(biotinoyl)-*N*′-(iodoacetyl) ethylenediamine (BIAM)-containing lysis buffer (50 mM Tris-HCl, pH 7.5, 150 mM NaCl, 0.5% Triton X-100, 10 *μ*g/ml aprotinin, and 10 *μ*g/ml leupeptin; the buffer was rendered free of oxygen by bubbling nitrogen gas at a low flow rate for 20 min). After sonication in a bath sonicator for three periods of 1 min each, the lysate was then clarified by centrifugation and subjected to immunoprecipitation with 1 *μ*g of TRPML1-specific Ab. Immunocomplexes labeled with BIAM were detected with HRP-conjugated streptavidin and developed with an enhanced chemiluminescence kit.

### 2.9. Fractionation and Western Blot Analysis

Total proteins from cultured cells were extracted on ice with lysis buffer (50 mM Tris-HCl, pH 8.0, 150 mM NaCl, 1 mM EDTA, 0.5% Nonidet P-40, and 0.01% protease inhibitor mixture) using the Nuclear/Cytosol Fractionation Kit for detecting TFEB. Proteins were separated by SDS-PAGE and transferred to nitrocellulose membranes. The membranes were blocked with 5% skim milk in Tris-buffered saline containing 0.1% Tween 20% (1x TBS-T) for 1 h at room temperature. Primary antibodies against LC3 (1 : 1000), p62 (1 : 10000), TRPML1 (1 : 1000), TFEB (1 : 1000), lamin B1 (1 : 1000), and *β*-actin (1 : 10000) were incubated overnight at 4°C. After washing with 1x TBS-T, the membranes were incubated with HRP-conjugated secondary antibodies for 1 h and developed using chemiluminescent substrates.

### 2.10. Transfection of siRNA

After treatment with M-CSF and RANKL for 40 h, BMMs were transfected with small interfering RNA (siRNA) against TRPML1 or with scrambled siRNA (scRNA) using Lipofectamine 3000. Further steps followed the method of Park et al. [4].

### 2.11. Statistical Analysis

All experiments were repeated at least three times. The data are expressed as mean ± standard deviation. Statistical analysis was performed by Student's *t*-test when two groups were compared or by one-way ANOVA followed by Bonferroni posttests when multiple groups were compared. Two-way ANOVA was used when two variables were analyzed. *p* value less than 0.05 was considered statistically significant.

## 3. Results

### 3.1. Lycorine Protects against LPS-Induced Bone Loss in Mice

**Figure 1:**
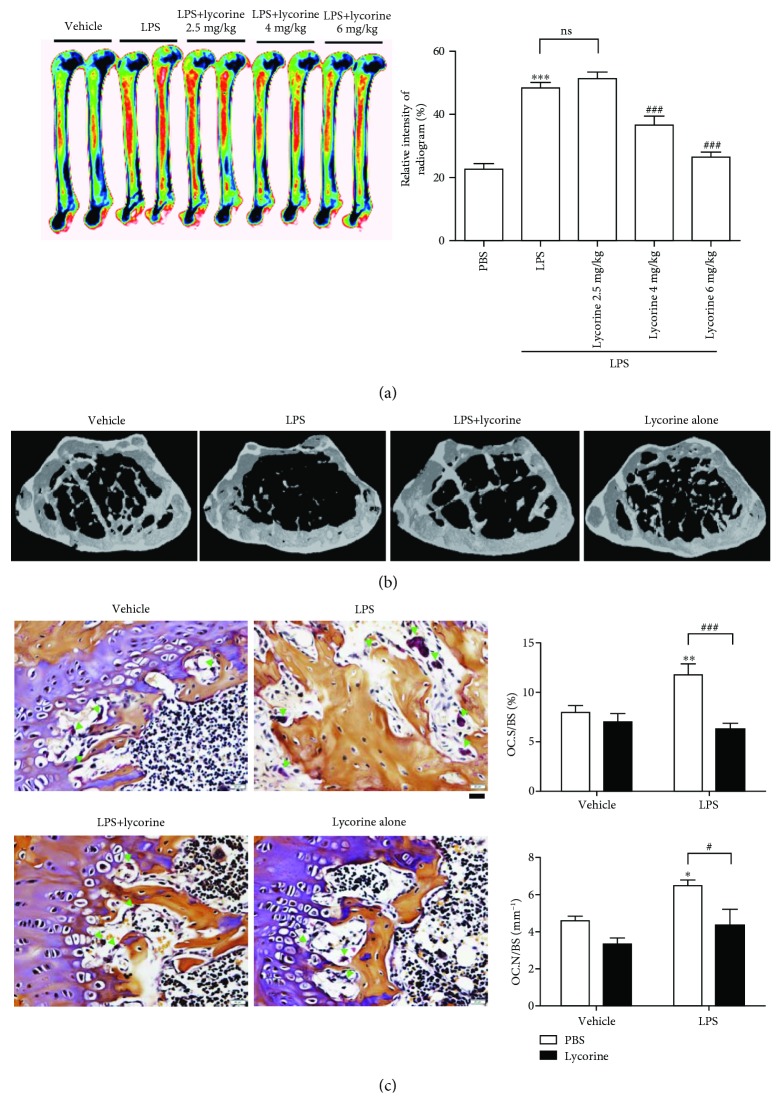
Lycorine protects against LPS-induced bone loss in mice. (a) Representative images and relative intensities of the distal femurs from X-ray radiograms of mice treated with PBS (*n* = 5), LPS (5 mg/kg/week) (*n* = 5), or LPS+lycorine (2.5 mg/kg/d, *n* = 5; 4 mg/kg/d, *n* = 5; 6 mg/kg/d, *n* = 6) were measured using the ImageJ program. ^∗^*p* < 0.05, ^∗∗^*p* < 0.01, and ^∗∗∗^*p* < 0.001 compared with PBS-treated mice. ^#^*p* < 0.05 and ^###^*p* < 0.001 compared with LPS-treated mice. Similar results were obtained in three independent experiments. (b) Representative *μ*CT images of distal femora 1.0 mm from the growth plate of mice treated with PBS (*n* = 5), LPS (5 mg/kg/week) (*n* = 5), LPS+lycorine (6 mg/kg/d) (*n* = 6), or lycorine only (6 mg/kg/d) (*n* = 5). Mice were treated for 3 weeks beginning at the age of 10 weeks. (c) To examine TRAP-positive OCs *in vivo*, mouse femora were excised, cleaned with a soft tissue, and decalcified in EDTA. Representative histological sections of the distal femoral metaphysis of mice from each of the 4 groups were stained for TRAP to identify OCs (indicated by arrows head) to calculate OC.N/BS (OC number divided by total bone surface) and OC.S/BS (OC surface area divided by total bone surface area). Scale bar: 20 *μ*m in the representative photos. ^∗^*p* < 0.05 and ^∗∗^*p* < 0.01 compared with PBS-treated mice. ^#^*p* < 0.05 and ^###^*p* < 0.001 compared with LPS-treated mice. Differences between groups were analyzed by two-way ANOVA, followed by Bonferroni posttests to compare the effect of lycorine (OC.S/BS and OC.N/BS; *p* < 0.01) and the effect of LPS (OC.N/BS; *p* < 0.01) and interaction (OC.S/BS; *p* < 0.05). Similar results were obtained in three independent experiments.

Lycorine has been shown to be anti-inflammatory [10], with ROS scavenging activity [11] as well as inhibition of autophagy [17]. Our previous results suggested that LPS induces bone loss by increasing autophagy *in vivo* [4] and LPS induced autophagy to enhance differentiation and activity of OCs by upregulating ROS [5]. This prompted us to hypothesize that lycorine attenuates LPS-induced bone loss in mice. To investigate the effect of lycorine on LPS-induced inflammatory bone loss, *μ*CT scans of femurs from mice treated with lycorine or vehicle after administration of LPS or PBS were analyzed. A 6 mg/kg dose of lycorine exhibited maximum protection compared to doses of 2.5 mg/kg and 4 mg/kg, respectively (Figure 1(a)). Body size or shape showed no change among the 4 groups when the mice were 13 weeks old. LPS induced significant bone loss with decreased bone mineral density (BMD), bone volume (BV/TV), and trabecular thickness (Tb.Th.) and increased trabecular space (Tb.Sp.) compared with PBS alone (Figure 1(b) and Table 1). The serum concentration of MCP-1 was also elevated upon LPS treatment (Table 1), indicating that LPS induced systemic inflammation. However, administration of lycorine with LPS attenuated the LPS-induced bone loss (Figure 1(b), Table 1). Treatment of lycorine with LPS increased BMD, BV/TV, and Tb.Th. compared with LPS alone, in addition to decreasing the enlargement of Tb.Sp. (Table 1), but lycorine alone did not exhibit any significant difference compared with vehicle (Figure 1(b), Table 1). As shown in Figure 1(c), OC.N/BS (the ratio of OC number to total bone surface area) and OC.S/BS (the ratio of OC surface area to total bone surface area) as assessed by *in vivo* TRAP staining were also significantly reduced when lycorine was injected in LPS-treated mice, indicating that lycorine reduced both number and size of OCs in LPS-treated mice. Consistent with these findings, serum CTX-1, a marker of bone resorption *in vivo* that was elevated by LPS treatment, was decreased when LPS-injected mice were treated with lycorine. However, cotreatment with lycorine did not significantly affect the levels of serum ALP and osteocalcin, which act as markers of bone formation *in vivo*, compared with LPS alone (Table 1). LPS-induced serum MCP-1 was also reduced by lycorine (Table 1), indicating that lycorine attenuated the systemic inflammation induced by LPS.

### 3.2. Lycorine Inhibits LPS-Induced OC Differentiation and OC Activity *In Vitro*

**Figure 2:**
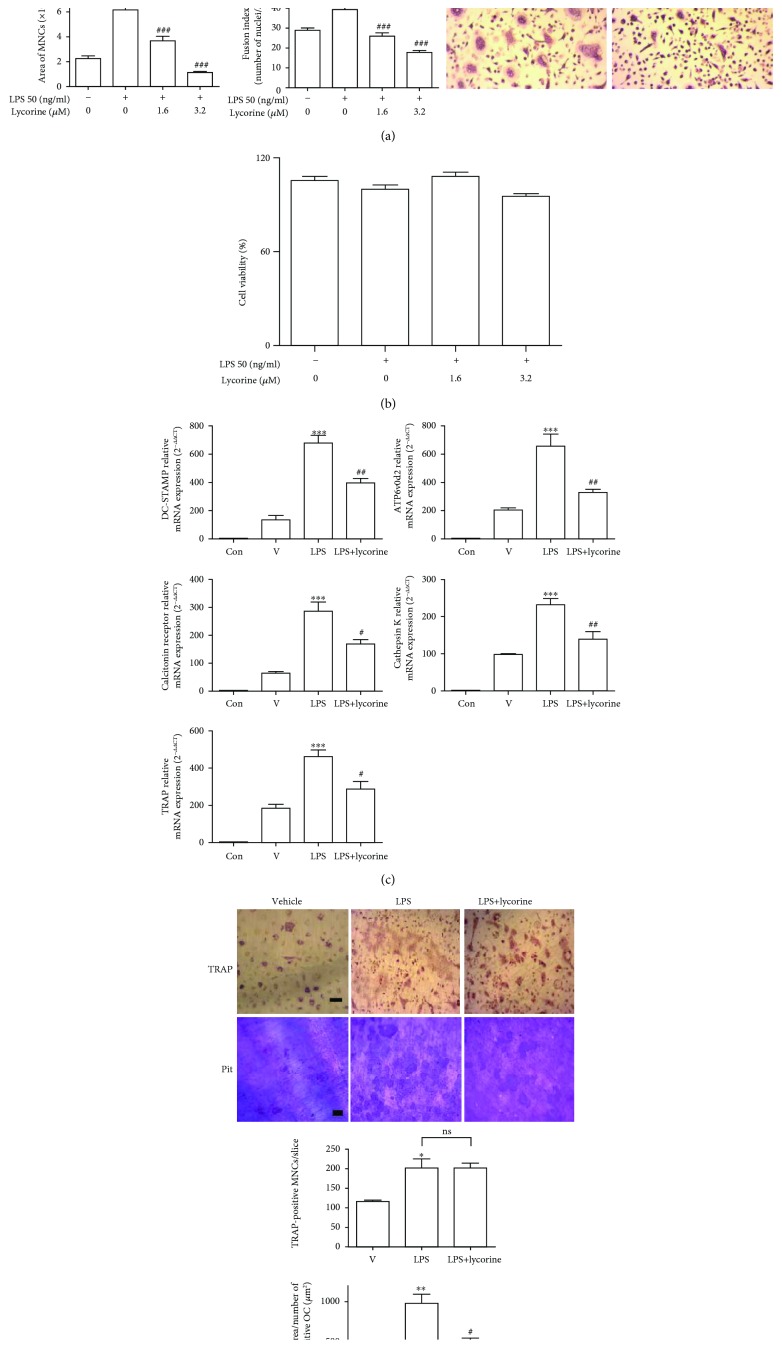
Lycorine inhibits LPS-induced OC differentiation and OC activity *in vitro.* BMMs (10^4^ cells/well) were prepared and incubated with RANKL (40 ng/ml) in the presence of M-CSF (30 ng/ml) for 40 h, washed, and then incubated further for 48 h (a, b, d) or 24 h (c) with LPS (50 ng/ml)±lycorine (1.6 *μ*M, 3.2 *μ*M) at the indicated concentration in the presence of M-CSF (30 ng/ml). LPS and lycorine were dissolved in PBS as a vehicle. Cells were fixed, and more than 70 TRAP-positive MNCs in each culture were randomly selected. The area, surrounded by a dotted line, and the maximum diameter, indicated by a double arrow of the formed OCs, were measured. The fusion index was presented as the average number of nuclei per TRAP-positive MNC formed in the culture. Representative photos are shown. Scale bar: 200 *μ*m in the representative OC photos (a). Cell viability was measured by MTT assay. No significant difference was found compared with PBS-treated cells (b). RNA from cells stimulated with LPS in the presence or absence of lycorine (1.6 *μ*M) was analyzed by qPCR. The expression level before RANKL pretreatment was set to 1 (c). Mature OCs were incubated further on whole dentine slices with M-CSF and LPS in the presence or absence of lycorine (1.6 *μ*M) for 4 d. After TRAP staining, the cells were removed, and the slices were stained with toluidine blue. Representative photos of TRAP-positive OCs and resorption pits are shown. Scale bar: 50 *μ*m in the representative photos. Total pit area/number of TRAP-positive OCs was calculated (d). ^∗^*p* < 0.05, ^∗∗^*p* < 0.01, and ^∗∗∗^*p* < 0.001 compared with PBS-treated pre-OCs. ^#^*p* < 0.05, ^##^*p* < 0.01, and ^###^*p* < 0.001 compared with LPS-treated cells. Similar results were obtained from three independent experiments.

Due to the contribution of OCs to protective effects of lycorine against LPS-induced bone loss, we assessed the effects of lycorine on LPS-stimulated OCs *in vitro*. RANKL-treated OC precursor cells were stimulated with LPS to cause the cells to differentiate into OCs, resulting in maximal OC formation after 48 h of exposure to LPS, as assessed by counting TRAP-positive MNCs. Our previous results demonstrated that LPS induces OCs to express the OC-specific genes TRAP, cathepsin K, DC-STAMP, ATP6v0d2, and calcitonin receptor [5]. Lycorine decreased TRAP-positive MNCs as well as the surface area and maximum diameter of OCs and the fusion index, which indicates the number of nuclei per OC (Figure 2(a)). Lycorine did not change the viability of BMMs under the assayed conditions (Figure 2(b)). Consistent with this result, the mRNA levels of TRAP, calcitonin receptor, cathepsin K, DC-STAMP, and ATP6v0d2 were markedly reduced in lycorine-treated cells (Figure 2(c)). Next, we examined whether lycorine attenuated *in vitro* bone resorption induced by LPS. Mature OCs generated from cells treated with lycorine in the presence of LPS showed significantly reduced total pit area/number of OCs compared to cells stimulated with LPS only (Figure 2(d)), indicating that lycorine inhibits OC activity.

### 3.3. Lycorine Decreases LPS-Induced Autophagy in OCs

**Figure 3:**
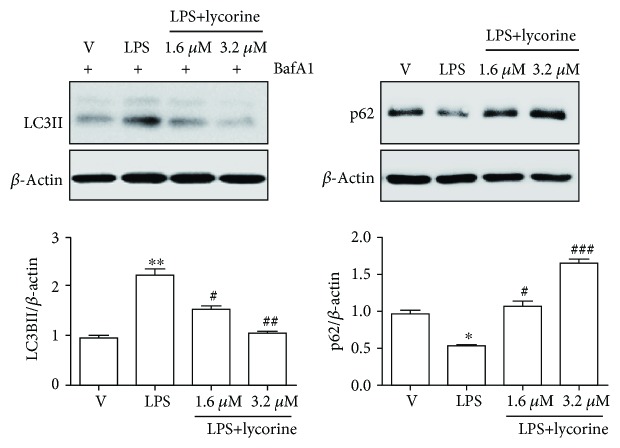
Lycorine decreases LPS-induced autophagy in OCs. BMMs were prepared and incubated with RANKL (40 ng/ml) in the presence of M-CSF (30 ng/ml) for 40 h, washed, and then incubated further for the indicated period with or without lycorine (1.6 *μ*M) in the presence of LPS (50 ng/ml) and M-CSF (30 ng/ml). The levels of LC3II and p62 were analyzed after 48 h of LPS stimulation with or without lycorine. Bafilomycin A1 (25 nM) was added 4 h before harvesting to stimulate the accumulation of LC3II. Quantification of LC3II and p62 normalized to *β*-actin was plotted. ^∗^*p* < 0.05 and ^∗∗^*p* < 0.01 compared with RANKL-pretreated cells treated with PBS (V). ^#^*p* < 0.05 and ^###^*p* < 0.001 compared with LPS-treated cells. Similar results were obtained in three independent experiments.

Excess autophagy has been reported to be responsible for inflammatory bone loss conditions such as rheumatic arthritis [23]. Since LPS induced autophagy to affect differentiation and activity of OC in our previous results [4, 5, 8], we hypothesized that lycorine inhibits autophagy to attenuate differentiation and activity in LPS-induced OCs. Therefore, we assessed whether lycorine decreased autophagy induced by LPS in OCs. Formation of autophagosomes was determined by immunoblotting cell lysates with an antibody against microtubule-associated protein light chain 3 (LC3) as a marker for activation of autophagic stimulus [24]. LPS increased the lipidated form of LC3 (LC3II), and addition of bafilomycin A1 led to accumulation of LC3II, whereas lycorine treatment significantly attenuated this accumulation (Figure 3). Degradation of p62/STSQM1 was evaluated as a surrogate marker for autophagic flux [25]. The expression level of p62 that was decreased by LPS stimulation was recovered by lycorine treatment in the presence of LPS (Figure 3).

### 3.4. Lycorine Reduces TFEB Nuclear Translocation by Attenuating Oxidation of TRPML1 via Decreased Mitochondrial ROS in OC

**Figure 4:**
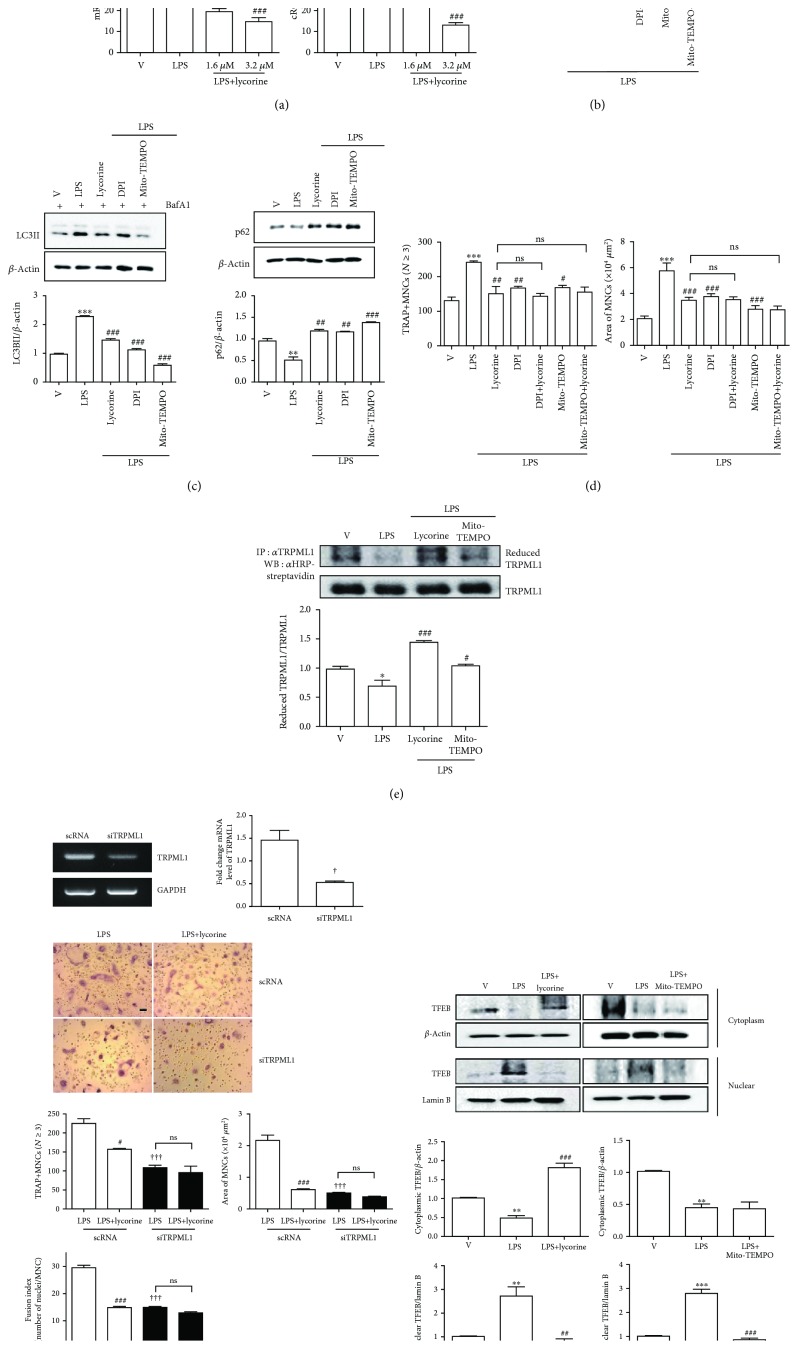
Lycorine reduces TFEB nuclear translocation by attenuating oxidation of TRPML1 via decreased mitochondrial ROS upon LPS stimulation in OC. BMMs were prepared, incubated with RANKL (40 ng/ml) in the presence of M-CSF (30 ng/ml) for 40 h, washed, and then incubated further with the indicated conditions (DPI, 5 nM; Mito-TEMPO, 100 nM; lycorine, 1.6 *μ*M) in the presence of LPS (50 ng/ml) and M-CSF (30 ng/ml). Mitochondrial ROS and cytosolic ROS were determined by flow cytometry using MitoSOX Red after 16 h and using H_2_DCF-DA after 24 h, respectively (a, b). After 48 h, cell lysates were prepared (c) or fixed (d). Cell lysates were subjected to Western blot to determine p62 and LC3II with the addition of bafilomycin A1 (25 nM) for 4 h. The quantified levels of p62 and LC3II are shown normalized to *β*-actin (c). Cell lysates were labeled with *N*-(biotinoyl)-*N*′-(iodoacetyl) ethylenediamine, and TRPML1 was immunoprecipitated (IP) from each sample. HRP-streptavidin immunoblotting was performed to evaluate the reduced form of TRPML1 (e). Cells were transfected with 50 nM of scRNA or siTRPML1 and incubated further for 48 h with LPS and M-CSF. siRNA-mediated silencing of TRPML1 was confirmed by RT-PCR and qPCR (f). After fixation, more than 70 TRAP-positive MNCs in each culture were randomly selected to determine the area and fusion activity of the formed OCs (d, f). Whole cell extracts, cytoplasmic fractions, and nuclear fractions were harvested from cultured cells and subjected to Western blot analysis with anti-TFEB Ab. Abs for *β*-actin and lamin B1 were used for the normalization of cytoplasmic and nuclear extracts, respectively. Quantification of TFEB normalized to *β*-actin or lamin B1 was plotted (g). ^∗^*p* < 0.05, ^∗∗^*p* < 0.01, and ^∗∗∗^*p* < 0.001 compared with RANKL-pretreated cells treated with PBS. ^#^*p* < 0.05, ^##^*p* < 0.01, and ^###^*p* < 0.001 compared with LPS-treated cells. ^†^*p* < 0.05 and ^†††^*p* < 0.001 compared with scRNA-treated cells. Similar results were obtained in three independent experiments.

Mitochondrial ROS have been reported to contribute to autophagy by oxidizing TRPML1, a lysosomal Ca channel [26], suggesting a critical role of ROS in inducing autophagy. Our previous results showed that LPS induces autophagy in OCs by stimulating cytoplasmic ROS (cROS) production [5]. That prompted us to hypothesize that lycorine decreased ROS to reduce LPS-induced autophagy in OCs. As we expected, lycorine treatment dramatically decreased mitochondrial ROS (mROS) and cROS after 16 h and 24 h LPS stimulation, respectively (Figure 4(a)). DPI, an inhibitor of NADPH oxidase, and Mito-TEMPO, an mROS scavenger, decreased mitochondrial ROS induced by LPS, and no further decrease was found when lycorine was added to DPI or Mito-TEMPO (Figure 4(b)). DPI and Mito-TEMPO decreased autophagic flux stimulated by LPS by decreasing LC3 II level and increasing p62 level (Figure 4(c)), supporting a role of ROS in LPS-induced autophagy. Lycorine did not further decrease the effect of DPI or Mito-TEMPO on area or number of OCs upon LPS stimulation (Figure 4(d)), indicating that the inhibitory activity of lycorine in OCs was mainly mediated by decreasing ROS levels induced by LPS. Since mROS has been reported to be responsible for transcription factor EB (TFEB) nuclear localization by oxidation of transient receptor potential cation channel, mucolipin subfamily (TRPML1) [26], we determined whether lycorine increased the reduced form of TRPML1. As shown in Figure 4(e), LPS decreased the level of reduced TRPML1, whereas lycorine treatment recovered the reduced form, as Mito-TEMPO did in the presence of LPS. To confirm the role of TRPML1 to exhibit the effect of lycorine on OCs, knockdown of TRPML1 was performed. TRPML1 silencing significantly reduced OC number, OC area, and fusion index that were increased by LPS (Figure 4(f)). No further decrease was found with lycorine treatment when TRPML1 was downregulated (Figure 4(f)), suggesting that TRPML1 is responsible for the inhibitory effect of lycorine in OCs. We determined whether lycorine delayed TFEB nuclear translocation stimulated by LPS as a next stage. As shown in Figure 4(g), LPS exposure increased the expression level of TFEB in the nucleus, whereas lycorine treatment significantly decreased it. Mito-TEMPO decreased nuclear TFEB as a positive control.

## 4. Discussion

We demonstrated that lycorine, a plant alkaloid purified from *Lycoris radiata* [9], protects mice from inflammatory bone loss induced by LPS. LPS injection led to a significant decrease in bone density with increased number and surface area of OCs with no change in bone formation, suggesting a critical role of OCs in LPS-induced bone loss. When lycorine was administered to LPS-injected mice, OC.N/BS, OC.S/BS, and serum CTX-1 levels were significantly reduced, whereas no changes were observed with *in vivo* bone formation markers, serum ALP and osteocalcin, implying that lycorine decreases bone loss induced by LPS in mice through OCs. *In vitro*, lycorine decreased TRAP-positive MNCs as well as the area and maximum diameter of OCs and fusion index that were increased by LPS. Bone resorption assessed in dentine slices was also decreased by lycorine. These results indicated that lycorine affected not only differentiation but also activity of OCs. Lycorine decreased LPS-induced autophagy when evaluated by decreased LC3II and increased p62. Our previous studies showed that LPS increased number and activity of OCs by inducing autophagy [4, 5]. These findings suggested that lycorine decreased number and activity of OCs by decreasing autophagy, finally leading to protection against bone loss.

We showed that lycorine decreased mROS, which act as a trigger signal for an axis of mROS/TRPML1/TFEB to induce autophagy. Lycorine also decreased cytoplasmic ROS induced by LPS. Our previous data exhibited that LPS increased the fusion of autophagosomes and autolysosomes [4] as well as autophagy induction by increasing cROS [5]. Blockade cROS by inhibition of NADPH oxidase induced by LPS decreased mROS, indicating that cROS also affected mitochondrial ROS. Many studies have demonstrated the association of ROS with autophagy, but the detailed mechanisms by which ROS induce autophagy are not completely explained. ATG4, which participates in the autophagy process, acts as a direct target of ROS [27], and ROS induce activation of NRF2 and FOXO3 as transcription factors to induce autophagy [28]. Increasing mitochondrial ROS has been reported to activate lysosomal TRPML1 channels, leading to nuclear translocation of TFEB and subsequent stimulation of autophagy [26], implying an important role of TRPML1 oxidation in TFEB nuclear translocation to induce autophagy. TRPML1, a lysosomal membrane protein, has been reported to play a role in autophagosome and lysosome fusion [29, 30] as well as acting as an ROS sensor [26]. Lysosome function and autophagy are modulated by an interaction between TRPML1 and TFEB [29–31], although there are other pathways for the activation of TFEB [30] such as mTOR inhibition. Our present study also shows that LPS induces mitochondrial ROS and subsequently oxidizes TRPML1 to induce TFEB nuclear translocation, suggesting that the effect of LPS on autophagy induction in OCs is regulated by an interaction loop of TRPML1 and TFEB. Removal of mROS by Mito-TEMPO reduced oxidized TRPML1 induced by LPS, suggesting that mROS participates in TRPML1 oxidation. Lycorine also decreased oxidized TRPML1 and TFEB nuclear translocation as well as mROS levels stimulated by LPS. Knockdown of TRPML1 decreased the effect of LPS and the inhibitory effect of lycorine on number of OC, OC area, and fusion index, suggesting that TRPML1 is required for the effects of LPS and lycorine on number and activity of OCs. Taken together, these results suggest that lycorine inhibits inflammatory bone loss by decreasing mROS that lead to decreased TFEB nuclear translocation by decreasing oxidation of TRPML1 in OCs.

Lycorine has been reported to have diverse pharmacological effects on various diseases with anti-inflammatory and antioxidant activity as well as downregulating autophagy with low toxicity and mild side effects [10, 11, 13, 14, 16, 17]. Our data demonstrated the protective effects of lycorine on inflammatory bone loss by attenuation of LPS-induced autophagy via an axis of mROS/TRPML1/TFEB, implying that decreasing oxidative stress in OCs is a potential therapeutic strategy for inflammatory bone loss.

## Figures and Tables

**Table 1:** Trabecular microarchitecture and biochemical markers of LPS with or without lycorine-treated mice.

	Vehicle (PBS)		LPS	
	PBS	Lycorine	PBS	Lycorine
BMD (mg/cm^3^)	213 ± 7	208 ± 3	147 ± 9^a^′′	183 ± 5^b^′
BV/TV (%)	17 ± 1	17.8 ± 0.3	12 ± 1^a^′′	15.3 ± 0.4^b^′′
Tb.Th (*μ*m)	74 ± 1	76 ± 2	57 ± 3^a^′′	69 ± 2^b^′′
Tb.Sp (*μ*m)	350 ± 11	356 ± 8	475 ± 30^a^′′	408 ± 8^b^
CTX (ng/ml)	26 ± 1	24 ± 3	51 ± 3^a^′′	24 ± 2^b^′′
ALP (U/l)	47 ± 1	41.1 ± 0.5	43 ± 5	33 ± 2
OCN (ng/ml)	24 ± 2	27 ± 1	26 ± 1	25 ± 1
MCP-1 (pg/ml)	150 ± 9	153 ± 11	279 ± 23^a^′	170 ± 34^b^′

PBS (*n* = 5); LPS (dissolved in PBS, 5 mg/kg) (*n* = 5); LPS+lycorine (dissolved in PBS, 6 mg/kg) (*n* = 6); lycorine (*n* = 5). Data are represented as mean ± SD. Differences between groups were analyzed by two-way ANOVA, followed by Bonferroni posttests to compare the effect of lycorine (BMD, *p* < 0.05; Tb.Th, *p* < 0.01; BV/TV and CTX-1, *p* < 0.001) and the effect of LPS (MCP-1, *p* < 0.05; BMD, Tb.Th, Tb.Sp, and CTX-1, *p* < 0.001) and interaction (BV/TV, Tb.Th, Tb.Sp, and MCP-1, *p* < 0.05; BMD, *p* < 0.01; CTX-1, *p* < 0.001). ^a^′*p* < 0.01 and ^a^′′*p* < 0.001 compared with vehicle-injected mice. ^b^*p* < 0.05, ^b^′*p* < 0.01, and ^b^′′*p* < 0.001 compared with LPS-injected mice.

## Data Availability

All data used to support the findings of this study are included within the article.
